# Does subjective socioeconomic status moderate the effect of basic psychological need satisfaction on undergraduates' affective forecasting?

**DOI:** 10.3389/fpsyg.2023.1227077

**Published:** 2023-07-14

**Authors:** Feng Zhang, Xiuzhen Jin, Linlin Fan, Yating Zhao, Meihua Sun, Xiaowei Geng

**Affiliations:** ^1^Jing Hengyi School of Education, Hangzhou Normal University, Hangzhou, China; ^2^Chinese Education Modernization Research Institute of Hangzhou Normal University (Zhejiang Provincial Key Think Tank), Hangzhou, China; ^3^Department of Child Education, Kunsan National University, Gunsan-si, Republic of Korea; ^4^School of Educational Science, Ludong University, Yantai, China; ^5^Institute for Education and Treatment of Problematic Youth, Ludong University, Yantai, China; ^6^Weihai Ocean Vocational College, Weihai, China

**Keywords:** affective forecast, self-determination theory, basic psychological needs (BPNS), subjective socioeconomic status (SES), impact bias

## Abstract

Affective forecasts are people's predictions of their future feelings in response to future events. In this study, based on the self-determination theory (SDT), we examined whether satisfying basic psychological needs influence undergraduates' affective forecasting and the moderating role of subjective socioeconomic status (SES). With a total of 423 undergraduate participants (177 males, 246 females), through one pilot study and three experiments, we first manipulated participants' basic psychological need satisfaction, i.e., autonomy need satisfaction (study 1), competence need satisfaction (study 2), and relatedness need satisfaction (study 3), then we asked low-SES and high-SES participants, respectively, to predict the pleasantness of a particular new product and evaluated the actual experience with the product. Results showed that the effect of basic psychological need on affective forecasting was not significant. When the need for autonomy need and competence need was satisfied, the impact bias was greater for the high SES than the low SES. Conversely, when the relatedness need was satisfied, the impact bias was greater for the low SES than the high SES. In conclusion, subjective SES moderated the influence of basic psychological needs satisfaction on increasing the impact bias in affective forecasting.

## 1. Introduction

People often want to know whether the things they are pursuing would make them happy or not. The ability to imagine possible future events allows people to anticipate the future hedonic consequences of decisions made in the here and now which is also known as affective forecasting. Affective forecasts are people's predictions of their future feelings in response to future events (Suddendorf and Busby, 2005; Gilbert and Wilson, 2007; Flynn et al., 2020). Affective forecasting plays a key role in guiding emotion regulation and decision-making (Loewenstein and Angner, 2003; Fellows, 2004; Lam et al., 2005; Kahneman, 2011). Therefore, it is important to understand whether people would make accurate affect forecasts.

A large number of studies have found that people are not capable of successfully predicting the duration and intensity of their emotional response to future events (Gilbert et al., 2004; Brown and McConnell, 2011; Morewedge and Buechel, 2013; Mata et al., 2018; Barber et al., 2023). People frequently overestimate how happy they will be after positive events and how sad they will feel after negative ones, which has been named the impact bias (Wilson et al., 2000; Wilson and Gilbert, 2003).

Previous research found some factors influencing affective forecasting, such as focusing illusion, i.e., people focus too much on the occurrence of the focal event and fail to consider the consequences of other events that are likely to occur (Wilson et al., 2000; Wilson and Gilbert, 2005; Lench et al., 2011), immune neglect, i.e., the tendency to overlook coping strategies and other aspects of the “psychological immune system” that can reduce future distress (Gilbert et al., 1998; Diener et al., 2006), aging (Barber et al., 2023), and motivation (Morewedge and Buechel, 2013; Pauketat et al., 2016; Geng and Jiang, 2017; Geng et al., 2018). However, based on our knowledge, few studies shed light on the influence of basic psychological needs (BPNS) on affective forecasting. In the present research, we addressed the influence of BPNS on affective forecasting by examining whether subjective socioeconomic status (SES) moderates this effect.

### 1.1. The relations between BPNS and affective forecasting

According to self-determination theory (SDT; Deci and Ryan, 2000), the satisfaction of three basic psychological needs fosters individual growth and wellbeing. Autonomy need refers to individuals taking actions on the basis of their internal self, which is essential for self-motivation and self-regulation (Deci and and Flaste, 1995). The need for autonomy is fulfilled by perceiving that one's activities are endorsed by or congruent with the self. Meanwhile, competence need entails believing in one's ability to control behaviors (Senécal et al., 2000). The need for competence is fulfilled by the experience that one can effectively bring about his/her desired effects and outcomes. Relatedness need refers to the need to love and be loved (Deci and and Flaste, 1995). The need for relatedness is fulfilled by feeling that one is close and connected to his/her significant others. The SDT posits that the fulfillment of these three needs is an essential criterion for optimal psychological functioning and wellbeing (Ryan et al., 2008). Compelling evidence shows that greater satisfaction with the needs for competence, autonomy, and relatedness is directly associated with intrinsic motivation and positive emotions in different contexts (e.g., Tang et al., 2020; Stanley et al., 2021). For example, a recent meta-analysis has shown positive associations between work-related need satisfaction and employee wellbeing (Van den Broeck et al., 2016). Based on these literature, we proposed that basic psychological needs satisfaction would also increase affective forecasting.

### 1.2. The moderating role of subjective SES

Although self-determination theory suggests that the three basic psychological needs (BPNS) are equivalent with regard to their importance for psychosocial functioning, individual differences in the need strength of the three basic psychological needs exist (Ryan and Deci, 2000). More recently, it has been forwarded that the existence of individual differences in need strength as moderators of relations between BPNS and outcomes (Van Hooff and De Pater, 2019; Wörtler et al., 2020).

Socioeconomic status (SES) is one of those factors which might lead to differences in the need strength of the three basic psychological needs. SES can be divided into two categories, i.e., objective SES and subjective SES. Objective SES measures an individual's financial resources, access to educational opportunities, and participation in social institutions (Oakes and Rossi, 2003). Subjective SES assesses social class rank relative to other members of the same university, community, or country, which usually is measured by the MacArthur Scale of subjective SES (Goodman et al., 2001), in which participants mark one of the 10 rungs on a ladder to indicate their own SES rank relative to comparison individuals. Previous studies (Kraus and Stephens, 2012) showed that subjective SES was more predictable for human behavior than objective SES. Therefore, we used subjective SES in the current research.

According to Stephens et al. (2014), individuals with the high SES tend to reflect and promote cultural norms of expressive independence, i.e., having independent selves, whereas those with the low SES tend to reflect and uphold the norms of hard interdependence, i.e., having interdependent selves. Individuals of high SES have a strong sense of control over their own lives (Markus and Kitayama, 1991; Lachman and Weaver, 1998; Johnson and Krueger, 2006), while low-SES individuals rely on good interpersonal relationships to obtain abundant social resources (Piff et al., 2010). Higher SES individuals tend to offer dispositional explanations of various social outcomes, while lower SES individuals tend to offer contextual explanations of social outcomes (Kraus et al., 2009). Thus, high-SES individuals may attach more importance to autonomy need and competence need than those in low SES. In contrast, individuals in the low SES may attach more importance to relatedness need than those in the high SES. In other words, individuals with high and low SES may be different in the need strength of three basic psychological needs. Specifically, high-SES individuals have higher need strength of competence and autonomy needs, whereas low-SES individuals have higher need strength of relatedness need.

Therefore, we propose that subjective SES would moderate the effect of basic psychological need satisfaction on affective forecasting. In specific, when autonomy need/competence need is satisfied, high-SES individuals would overestimate their affective experience on products that are related to autonomy need/competence need more than low-SES individuals, i.e., show greater impact bias on the product. In contrast, when relatedness need is satisfied, low-SES individuals would overestimate their affective experience on products that are related to relatedness need more than high-SES individuals, i.e., show greater impact bias on the product.

### 1.3. The current research

Our primary objective was to show that the predicted relations between BPNS and affective forecasting would vary as a function of undergraduates' subjective SES. Specifically, when the autonomy need/competence need was satisfied, high-SES undergraduates would show greater impact bias in affective forecasting than low-SES undergraduates; conversely, when the relatedness need was satisfied, low-SES individuals would show greater impact bias in affective forecasting than high-SES individuals.

To achieve this objective, we have conducted four studies. First, in the pilot study, we tested the relative importance of three basic psychological needs for high-SES individuals and low-SES individuals, respectively. Then, we examined the interactive effect of basic psychological need satisfaction and SES on affective forecasting through three studies. In the pilot study, we expected that high-SES individuals attached more importance to competence need and autonomy need than low-SES individuals, whereas low-SES individuals attached more importance to relatedness need than high-SES undergraduates.

In three experiments, we examined the effect of autonomy need fulfillment (experiment 1), competence need fulfillment (experiment 2), and relatedness need fulfillment (experiment 3) on the impact bias of the low-SES individuals and high-SES individuals, respectively. We expected that when autonomy need/competence need was satisfied, high-SES undergraduates would overestimate their affective experience on products that are related to autonomy need/competence need more than low-SES undergraduates. In contrast, when relatedness need was satisfied, low-SES individuals would overestimate their affective experience on products that are related to relatedness need more than high-SES individuals. The project was reviewed and approved by the Academic Ethics Committee of the School of Education at the University before being conducted.

## 2. Pilot study: the relative importance of three basic psychological needs for high-SES and low-SES individuals, respectively

The goal of the present study was to examine the relative importance of three basic psychological needs for high-SES individuals and low-SES individuals, respectively. According to the self-determination theory, psychological needs have three basic types, i.e., need for autonomy, competence, and relatedness (Deci and Ryan, 2000). However, the relative importance of the three basic needs may be different for the low SES and the high SES, respectively. Therefore, in this pilot experiment, the task of dividing the circular area was used to explore the relative importance of the three basic psychological needs for the low SES and the high SES, respectively.

### 2.1. Methods

#### 2.1.1. Participants

For the study, 63 college students (30 males and 33 females, *M*_age_ = 21.73 years, *SD* = 1.86) were selected as participants from a university in China.

#### 2.1.2. Design and procedure

First, SES was manipulated. Participants were randomly assigned to complete a low or high-SES manipulation (Kraus et al., 2010; Piff et al., 2012), in which they were shown an image of a ladder consisting of 10 rungs and asked to either make a direct comparison between themselves and people who are relatively better off (low-SES condition) or worse off (high-SES condition). According to Cheon and Hong (2017), participants were provided with the following instructions:

*Think of this ladder as representing where people stand in China. Now, please compare yourself to the people at the very bottom (top) of the ladder. These are the people who are the worst (best) off—those who have the least (most) money, least (most) education, and least (most) respected jobs. In particular, we'd like you to think about how YOU ARE DIFFERENT FROM THESE PEOPLE in terms of your own income, educational history, and job status. Where would you place yourself on this ladder relative to these people at the very bottom (top)? Please select the number that corresponds to the rung where you think you stand in relation to these people*.

To strengthen the SES manipulation effect, after selecting a rung, participants were then instructed to write a description of what it would be like to have an interaction with the person they had just compared themselves with. Participants were provided with the following instructions:

*Now imagine yourself in a getting acquainted interaction with one of the people you just thought about from the very bottom (top) of the ladder. Think about how the DIFFERENCES BETWEEN YOU might impact what you would talk about, how the interaction is likely to go, and what you and the other person might say to each other. Please write a brief description about how you think this interaction would go*.

Then, to test whether the manipulation of SES was successful, participants were also asked to complete the MacArthur Scale of subjective SES (Goodman et al., 2001), in which participants marked one of the 10 rungs on a ladder to indicate their own SES rank. The self-reported MacArthur Scale scores of low-SES condition and high-SES condition were compared and found there was a significant difference between participants in low-SES condition and high-SES condition, *M*_highSES_ = 5.10, *SD* = 1.74, *M*_lowSES_ = 3.84, *SD* = 1.82, *t* (61) = 2.80, 95% CI [0.36, 2.14], *p* < 0.01, and *d* = 0.72, which indicated that the manipulation of the subjective SES in the pilot study was successful.

Finally, to measure the relative importance of basic psychological needs, the participants were first introduced to the definitions of three basic psychological needs, i.e., “Autonomy need refers to individuals taking actions on the basis of their internal self; competence need entails believing in one's ability to control behaviors; relatedness need refers to the need to love and be loved,” and then asked to divide the area of a circle of 12.56 square centimeters into three pieces, just like cutting a cake into three pieces with each piece representing one basic psychological need, according to the relative importance of the three needs to themselves. A larger area of a certain basic psychological need entailed that this need was more important than the others.

### 2.2. Results

**Figure 1:**
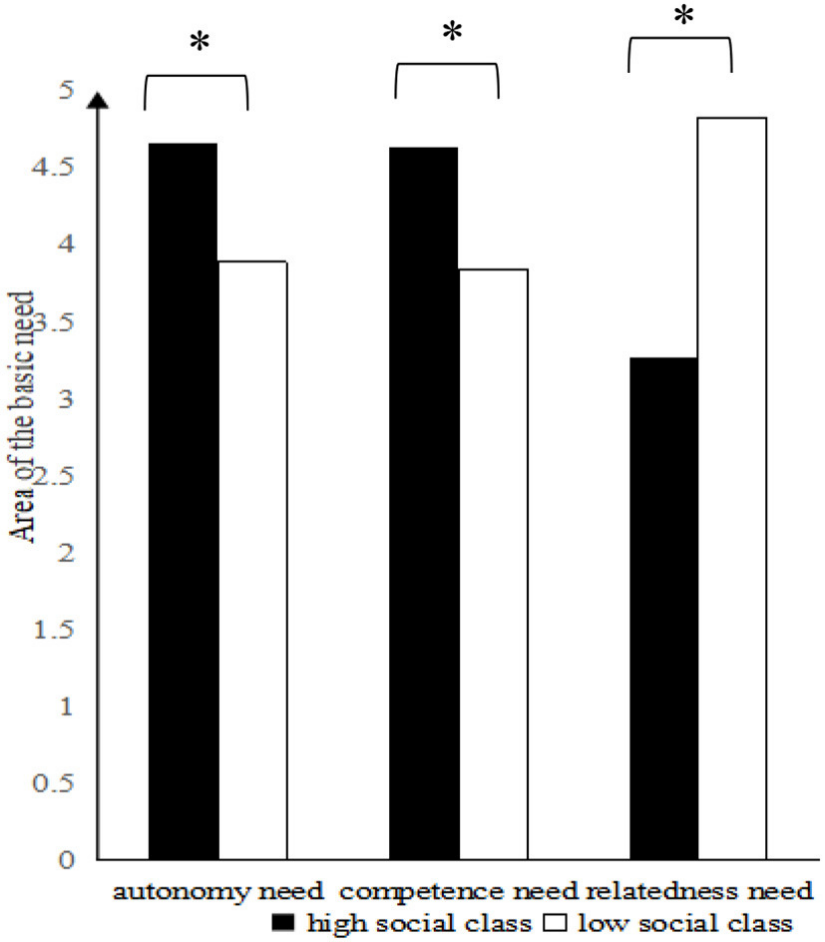
Basic psychological needs for high-SES individuals and low-SES individuals. ^*^p < 0.05.

To test the differences in importance among the three basic psychological needs for the high SES and the low SES, independent sample *T*-tests were conducted. From Figure 1, we can see that for autonomy need, high SES individuals attached more importance than those in the low SES (*M*_highSES_ = 4.66, *SD* = 1.51, *M*_lowSES_ = 3.89, *SD* = 1.53, *t* (61) = 2.01, 95% CI [0.01, 1.54], *p* = 0.04, *d* = 0.52); for competence need, high-SES individuals attached more importance than those in the low SES (*M*_highSES_ = 4.64, *SD* = 1.29, *M*_lowSES_ = 3.84, *SD* = 1.59, *t* (61) = 2.17, 95% CI [0.06, 1.52], *p* = 0.03, *d* = 0.56); for relatedness, low-SES individuals attached more importance than those in the high SES (*M*_highSES_ = 3.27, *SD* = 1.43, *M*_lowSES_ = 4.83, *SD* = 2.17, *t* (61) = −3.36, 95% CI [−2.49, −0.63], *p* = 0.01, *d* = −0.86). Results indicated that high-SES individuals attached more importance to autonomy need and competence need than low-SES individuals, whereas low-SES individuals attached more importance to relatedness need than high-SES individuals.

### 2.3. Discussion

The pilot study found that high-SES individuals attached more importance to autonomy need and competence need, whereas low-SES individuals attached more importance to relatedness need. These outcomes were consistent with our hypothesis. High-SES individuals had a stronger sense of control over their own lives and tended to have more independent choices (Lachman and Weaver, 1998; Johnson and Krueger, 2006). Thus, these individuals would have stronger competence need and autonomy need. Low-SES individuals developed interdependent selves (Stephens et al., 2014) and then would have stronger relatedness need.

## 3. Study 1: autonomy need satisfaction and affective forecasting

Based on the result of the pilot study, study 1 aims to examine whether high-SES individuals would have a greater impact bias in affective forecasting on the product which could satisfy their autonomy need more than low-SES individuals.

### 3.1. Methods

#### 3.1.1. Participants

A power analysis using G^*^Power 3.1 (Faul et al., 2009) suggested a sample size of 120 participants that would provide 80% power to detect medium interaction effects (*f* = 0.30). A total of 120 college students (45 males and 75 females, *M*_age_ = 18.13 years, *SD* = 0.70) from a university in China participated in this study.

#### 3.1.2. Experimental design and materials

A 2 (subjective SES: high SES vs. low SES) × 2 (autonomy need satisfaction: yes vs. no) between-subject design was used.

##### 3.1.2.1. Manipulation of the subjective SES was the same as that in the pilot study

Significant differences were observed in the self-reported scores on the MacArthur Scale, *M*_highSES_ = 5.30, *SD* = 1.48, *M*_lowSES_ = 4.68, *SD* = 1.46, *t* (118) = 2.30, 95% CI [0.09, 1.15], *p* < 0.05 and *d* = 0.42. The results indicated that the manipulation of the subjective SES in the current study was successful.

##### 3.1.2.2. Manipulation of autonomy need satisfaction

We selected Frey chocolates as the material because it was a Swiss brand with which the participants were very unfamiliar. All the participants had not eaten Frey chocolate before. In fact, only 2% of participants had heard of the brand. Thus, they had no previous experience with the brand. Therefore, the influence of previous experience on affective forecasting of the product was excluded.

When participants entered the lab, they would see three chocolates on the table that looked the same but had different flavors (i.e., air, orange, and milk). Under the condition of the autonomy need being satisfied, the participants selected the flavor of chocolates by themselves. However, under the condition in which their autonomy need was not satisfied, the flavor of chocolate was selected by the conductor of the experiment. Participants in the non-autonomy condition were assigned the same chocolate by the experimenter as what participants chose in the autonomy condition to ensure that the participants in the autonomy condition and the non-autonomy condition tasted the same flavor of chocolates.

Under the condition of more autonomy need satisfaction, the participants were told, “*Here are three chocolates from Frey, which is a famous Swiss brand. There are three different flavors, that is, air, orange, and milk. You can choose the one you like best and savor it*.” Under the condition of non-autonomy need satisfaction, the experimenter chose the participants. The participants were told, “*Here are three chocolates from Frey, which is a famous Swiss brand. There are three different flavors, that is, air, orange, and milk. As the experimenter, I would like you to savor this one*.”

##### 3.1.2.3. Affective forecasting measure

The affective forecasting of the chocolate was measured by asking participants to predict how pleasant the taste of the chocolate would be and how much they would like the chocolate. Responses were made on a 5-point scale ranging from 1 (completely unpleasant/dislike) to 5 (completely pleasant/like). The average of two items was calculated as affective forecasting.

##### 3.1.2.4. Affective experience measure

The affective experience with the chocolate was measured by asking participants to assess how pleasant the aftertaste of the chocolate was and how much they liked the particular chocolate. Responses were made on a 5-point scale ranging from 1 (completely unpleasant/dislike) to 5 (completely pleasant/like). The average of two items was calculated as affective experience.

#### 3.1.3. Procedure

All participants were first randomly assigned into two groups: high SES and low SES. Upon entering the experimental room outfitted with a table and two chairs, the participant was seated at the table. On the table were three chocolates. The participants were asked to select one by themselves or assigned by the experimenter. Once the participants chose a flavor or were assigned a flavor, the other two chocolates would be removed from the table with only the chosen one left. Before tasting the chocolate, the participants were asked to predict their emotions on tasting the selected flavor of chocolate. After tasting, the participants were asked to evaluate the actual affective experience of savoring the chocolate.

### 3.2. Results

According to Gilbert et al. (1998), the difference in affective forecasting minus affective experience was taken as the index of impact bias.

**Figure 2:**
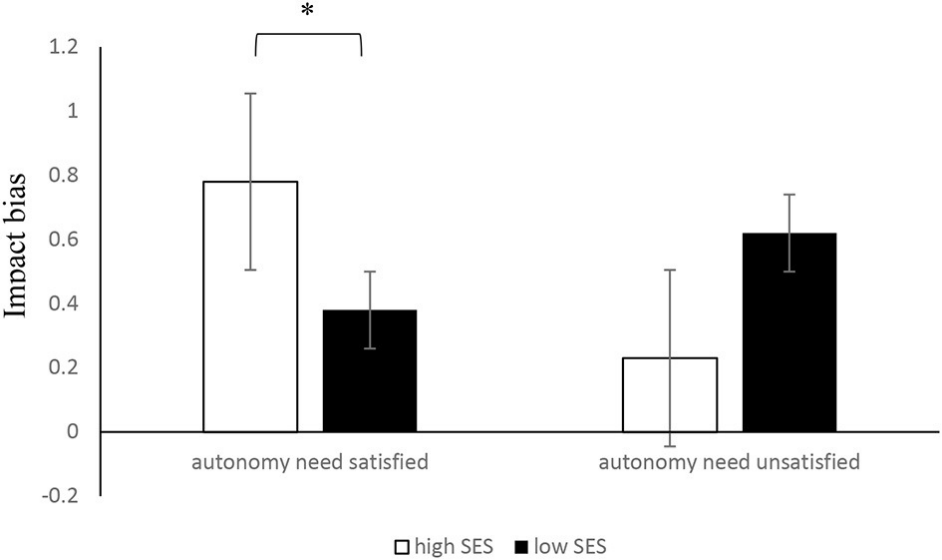
Interactive effect of autonomy need satisfaction and SES on impact bias. ^*^p < 0.05.

As for impact bias, the results of the 2 (subjective SES: high SES vs. low SES) × 2 (autonomy need satisfaction: yes vs. no) ANOVA revealed that the main effect of subjective SES on impact bias was not significant; *F* (1, 119) = 0.01, *p* = 0.97, and η^2^ = 0.01. The main effect of autonomy need was not significant; *F* (1, 119) = 0.69, *p* = 0.41, and η^2^ = 0.01. Meanwhile, the interaction of subjective socioeconomic status and autonomy need on impact bias was significant; *F* (1, 119) = 4.19, *p* = 0.04, and η^2^ = 0.04. Moreover, a simple effect analysis showed that, in the case of high-SES individuals, the individual whose autonomy need was satisfied had a larger impact bias than the individual whose autonomy need was not satisfied, which was marginally significant with a large effect size, i.e., *M*_satisfied_ = 0.78, *SD* = 1.09, *M*_unsatisfied_ = 0.23, *SD* = 1.17, *t* (58) = 1.88, 95% CI [−0.03, 1.13], *p* = 0.06 and *d* = 0.96. In the case of low-SES individuals, no significant difference was observed on whether the autonomy need was satisfied more or less; *M*_satisfied_ = 0.38, *SD* = 0.69, *M*_unsatisfied_ = 0.62, *SD* = 1.16, *t* (58) = −0.94, 95% CI [−0.73, 0.26], *p* = 0.35, and *d* = −0.25. When the autonomy need was satisfied, the high-SES individuals had a larger impact bias than the low-SES individuals, *M*_highSES_ = 0.78, *SD* = 1.09, *M*_lowSES_ = 0.38, *SD* = 0.69, *t* (58) = 1.7, 95% CI [−0.07, 0.87], *p* = 0.09 and *d* = 0.44. When the autonomy need was not satisfied, no significant difference was observed between high-SES individuals and low-SES individuals; *M*_highSES_ = 0.23, *SD* = 1.17, *M*_lowSES_ = 0.62, *SD* = 1.16, *t* (58) = −1.27, 95% CI [−0.98, 0.22], *p* = 0.21 and *d* = −0.33. The interaction is shown in Figure 2.

### 3.3. Discussion

Results of study 1 showed that when autonomy need was satisfied more, the impact bias of high-SES individuals was greater than that of low SES. In contrast, when autonomy need was satisfied less, no significant difference was observed between high-SES and low-SES participants, thus supporting our hypothesis.

Given that individuals in the high SES attached more importance to autonomy need satisfaction than low-SES individuals, high-SES individuals would have more positive expectations for the experience (i.e., savoring the flavor of Frey chocolate) when it was chosen by themselves. Hence, these individuals would overestimate their own positive emotions more.

Study 2 will further study the interactive effect of competence need satisfaction and subjective SES on affective forecasting.

## 4. Study 2: competence need satisfaction and affective forecasting

The goal of study 2 was to examine whether high-SES individuals would show greater impact bias in affective forecasting on products than those in the low SES when competence need was satisfied. In study 2, an English word test was used to manipulate the satisfaction of competence need. Under the condition of competence need satisfaction, the participants were given feedback that their correct rate on the test was higher than the normal correct rate of college students. In contrast, under the condition of competence need non-satisfaction, the participants were told that their correct rate in the test was lower than the normal correct rate of college students. After the manipulation, they predicted the pleasantness of using a new English word book for the College English Test Band 6 (CET-6) and evaluated their experience after using the book. We predicted that when their competence needs were satisfied (i.e., high English level in English test), high-SES individuals would show greater impact bias in predicting the pleasantness of using the new English word book than those in the low-SES individuals.

### 4.1. Methods

#### 4.1.1. Participants

For the study, we conducted sample size estimation using G^*^Power 3.1 (Faul et al., 2009) to determine the sufficient number of participants needed to detect a reliable effect. A power analysis suggested a sample size of 120 participants that would provide 80% power to detect medium interaction effects (*f* = 0.30). Finally, 120 college students (55 males and 65 females, *M*_age_ = 18.15 years, *SD* = 0.69) were selected as participants from a university in China.

#### 4.1.2. Experimental materials and design

A 2 (subjective SES: high SES vs. low SES) × 2 (competence need satisfaction: yes vs. no) between-subject design was used.

##### 4.1.2.1. Manipulation of the subjective SES was the same as that in the pilot study

Significant differences were observed in the self-reported scores on the MacArthur Scale, i.e., *M*_highSES_ = 5.32, *SD* = 1.45, *M*_lowSES_ = 4.61, *SD* = 1.66, *t* (118) = 2.49, 95% CI [0.28, 0.14], *p* < 0.05 and *d* = 0.46, which indicated that the manipulation of subjective SES was successful.

##### 4.1.2.2. Manipulation of competence need satisfaction

Participants took part in an English word test first and were given feedback on their performance. To manipulate the competence need satisfaction, one group of participants was told that the normal correct rate of college students was 60%, which was lower than their performance and then satisfy their competence needs, and the other group of participants was told that the normal correct rate of college students was 80%, which was higher than their performance and then could not satisfy their competence needs.

To test the manipulation of competence need satisfaction, the participants were asked to evaluate their English level after the English word test: 1 = very low to 5 = very high. Significant differences were noted in self-reported English competence, *M*_satisfied_ = 3.46, *SD* = 0.62, *M*_unsatisfied_ = 2.50, *SD* = 0.65, *t* (118) = 8.59, 95% CI [0.76, 1.23], *p* < 0.05 and *d* = 0.46, In addition, to exclude the effect of this manipulation on participants' emotion and self-esteem, the participants were asked to report their emotions and self-esteem after the English word test as well. No significant differences were observed in the self-reported emotion (*M*_satisfied_ = 3.98, *SD* = 0.80, *M*_unsatisfied_ = 3.70, *SD* = 0.94, *t* (118) = 1.75, 95% CI [-0.04, 0.59], *p* = 0.08) and self-esteem (*M*_satisfied_ = 17.66, *SD* = 3.41, *M*_unsatisfied_ = 18.79, *SD* = 3.84, *t* (118) = −1.70, 95% CI [−2.44, 1.19], *p* = 0.09), which demonstrated that the manipulation of competence need satisfaction was successful.

##### 4.1.2.3. Affective forecasting measure

A new English word book for the CET-6 exam, which included English crosswords, was used for affective forecasting. All participants were freshmen who had just entered the university and had not been exposed to CET-6. Therefore, the influence of previous experience on affective forecasting of the product was excluded.

The participants were introduced to the English word book first and then asked to predict how much they would like the book and how pleasant the experience of using the material would be. Responses were made on a 5-point scale ranging from 1 (completely dislike/unpleasant) to 5 (completely like/pleasant).

##### 4.1.2.4. Affective experience measure

The affective experience of the English word book was measured by asking the participants to assess how much they liked the book and how pleasant their experience of using the book was. Responses were made on a 5-point scale ranging from 1 (completely dislike/unpleasant) to 5 (completely like/pleasant).

#### 4.1.3. Procedure

Upon entering the laboratory, all participants were first randomly assigned into two groups: the high-SES group and the low-SES group. First, the participants were asked to finish the English word test and were given feedback to manipulate the competence need satisfaction. Then, the participants were introduced to the English word book and asked to answer the affective forecasting questionnaire. After using the word book for 10 min, the participants were asked to answer the affective experience questionnaire.

### 4.2. Results

**Figure 3:**
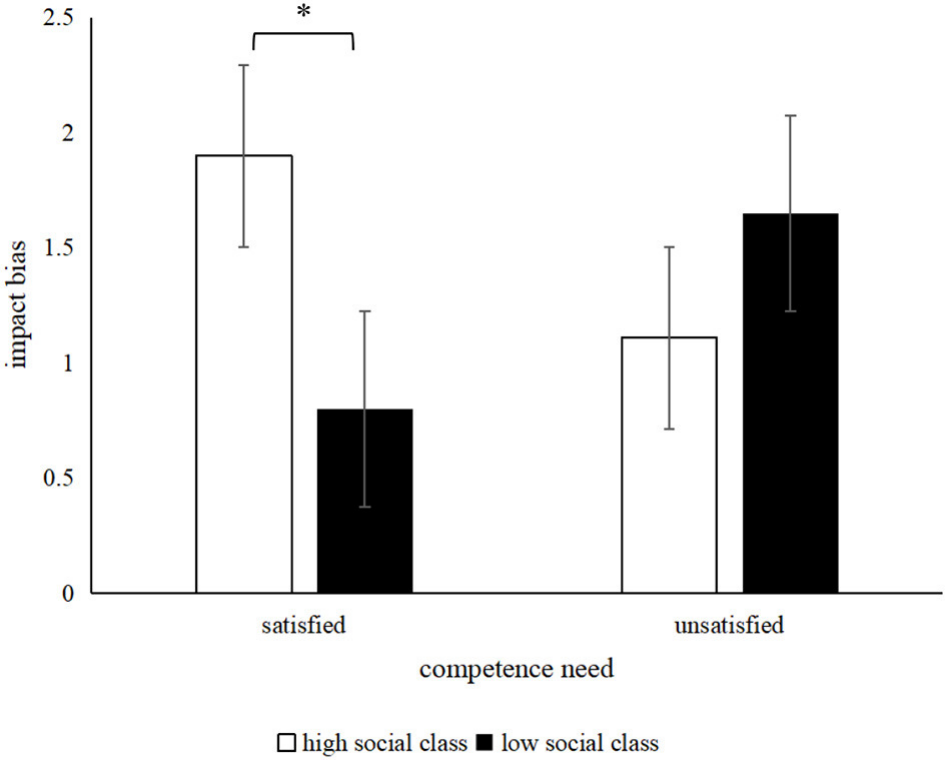
Interactive effect of competence need satisfaction and SES on impact bias. ^*^p < 0.05.

According to Gilbert et al. (1998), the difference in affective forecasting minus affective experience was taken as the index of impact bias. As for affective impact bias, the results of the 2 (subjective SES: high SES vs. low SES) × 2 (competence need satisfaction: yes vs. no) ANOVA revealed that the main effect of subjective SES on impact bias was not significant, i.e., *F* (1, 119) = 0.72, *p* = 0.40, and η^2^ = 0.01. Meanwhile, the main effect of competence need was not significant; *F* (1, 119) = 0.01, *p* = 0.93, and η^2^ = 0.01. The interaction of the subjective SES and competence need satisfaction on impact bias was significant, i.e., *F* (1, 116) = 6.09, *p* = 0.02, and η^2^ =.05. A simple effect analysis showed that, when the competence need was satisfied, the high-SES individuals had a larger impact bias than the low-SES individuals, *M*_highSES_ = 1.90, *SD* = 1.95, *M*_lowSES_ = 0.80, *SD* = 1.75, *t* (57) = 2.27, 95% CI [0.13, 2.06], *p* = 0.03 and *d* = 0.60. When the competence need was not satisfied, no significant difference was observed between high-SES individuals and low-SES individuals; *M*_highSES_ = 1.11, *SD* = 1.78, *M*_lowSES_ = 1.64, *SD* = 1.74, *t* (59) = −1.18, 95% CI [−1.44, 0.37], *p* = 0.24 and *d* = −0.31. The interaction is shown in Figure 3.

### 4.3. Discussion

Results of study 2 showed that when competence need was satisfied, the impact bias of high-SES individuals was greater than that of low SES. In contrast, when competence need was not satisfied, no significant difference was observed between high-SES and low-SES participants, thus supporting our hypothesis. As individuals in the high SES attached more importance to competence need satisfaction than those in the low SES, high-SES individuals would have more positive expectations for the experience (i.e., using the new type of English word book) when their competence need was satisfied, thus overestimating their own positive emotions more.

Study 3 will further study the interactive effect of relatedness need satisfaction and subjective SES on impact bias in affective forecasting.

## 5. Study 3: relatedness need satisfaction and affective forecasting

The goal of study 3 was to examine whether the high-SES individuals would show a smaller impact bias in affective forecasting than those in the low SES when relatedness need was satisfied. In study 3, peer nomination was used to manipulate the satisfaction of relatedness need. Under the condition of relatedness satisfaction, the participants were given feedback that their numbers of nominations were higher than the average number of nominations in their class. In contrast, under the condition of relatedness non-satisfaction, the participants were told that their numbers of nominations were lower than the average number of nominations in their class. After the manipulation, they predicted the pleasantness of playing an interactive game named German Heart Disease and evaluated their experience after the game. We predicted that when their relatedness need was satisfied, high-SES individuals would show a smaller impact bias in predicting the pleasantness of playing the interactive game than low-SES individuals.

### 5.1. Methods

#### 5.1.1. Participants

For the study, we conducted sample size estimation using G^*^Power 3.1 (Faul et al., 2009) to determine the sufficient number of participants needed to detect a reliable effect. A power analysis suggested a sample size of 120 participants that would provide 80% power to detect medium interaction effects (*f* = 0.30). Finally, 120 college students (47 males and 73 females, *M*_age_ = 18.15 years, *SD* = 0.70) were chosen as participants from a university in China.

#### 5.1.2. Experimental design and materials

A 2 (subjective SES: high SES vs. low SES) × 2 (relatedness need satisfaction: yes vs. no) between-subject design was used.

##### 5.1.2.1. The manipulation of the subjective SES was the same as that in the pilot study

Significant differences were observed in the self-reported scores on the MacArthur Scale, *M*_highSES_ = 5.12, *SD* = 1.71, *M*_lowSES_ = 4.48, *SD* = 1.48, *t* (118) = 2.17, 95% CI [0.06, 1.21], *p* = 0.03 and *d* = 0.40, which indicated that the subjective SES was manipulated successfully.

##### 5.1.2.2. Manipulation of relatedness need

Peer nomination was adopted to manipulate the relatedness need satisfaction. All the participants were asked to nominate three persons with whom they want to join in the school outing. After nominating, the participants were given feedback on their own numbers of being nominated personally by the computer. To manipulate participants' relatedness need satisfaction, one group of participants were informed that their nominations were higher than the average number of nominations, which satisfied relatedness needs, the other group were informed that their nominations were lower than the average number of nomination, which did not satisfy relatedness needs.

To test the manipulation of relatedness need satisfaction, the participants were asked to evaluate their popularity after peer nomination: 1 = not at all to 5 = very popular. Significant differences were noted in self-reported popularity, *M*_satisfied_ = 3.65, *SD* = 0.66, *M*_unsatisfied_ = 3.12, *SD* = 0.69, *t* (118) = 4.33, 95% CI [0.29, 0.78], *p* = 0.01 and *d* = 0.80. In addition, to exclude the effect of this manipulation on participants' emotion and self-esteem, the participants were asked to report their emotions and self-esteem after the peer nomination as well. No significant differences were observed in emotion (*M*_satisfied_ = 3.78, *SD* = 0.61, *M*_unsatisfied_ = 3.60, *SD* = 0.64, *t* (118) = 1.60, 95% CI [-0.04, 0.41] and *p* = 0.11) and self-esteem (*M*_satisfied_ = 17.37, *SD* = 3.83, *M*_unsatisfied_ = 18.17, *SD* = 4.18, *t* (118) = −1.09, 95% CI [−2.25, 0.65] and *p* = 0.28), which demonstrated that the manipulation of relatedness need satisfaction was successful.

##### 5.1.2.3. Affective forecasting measure

An interactive board game called German Heart Disease, which was a relatively new game, was adopted as the experimental material. To exclude the influence of previous experience on the preference for the board game, 30 students were randomly selected from a university for a preliminary experiment to investigate their previous knowledge of the board game. All college students mentioned that they had not played the board game before.

Participants were introduced to German Heart Disease first and were asked to predict how much they would like the game and how pleasant the experience of the game would be. Responses were made on a 5-point scale ranging from 1 (completely dislike/unpleasant) to 5 (completely like/pleasant).

##### 5.1.2.4. Affective experience measure

The affective experience of German Heart Disease was measured by asking participants to assess how much they liked the game and how pleasant the experience was. Responses were made on a 5-point scale ranging from 1 (completely unlike/unpleasant) to 5 (completely like/pleasant).

#### 5.1.3. Procedure

Upon entering the laboratory, all participants were first randomly divided into two groups: the high-SES group and the low-SES group. First, the participants were asked to finish the peer nomination task on the computer and were given feedback to manipulate the relatedness need satisfaction. Then, participants were introduced to the board game and were asked to complete the affective forecasting questionnaire. All participants played the board game with the confederate. After playing the game, the participants were asked to answer the affective experience questionnaire.

### 5.2. Results

**Figure 4:**
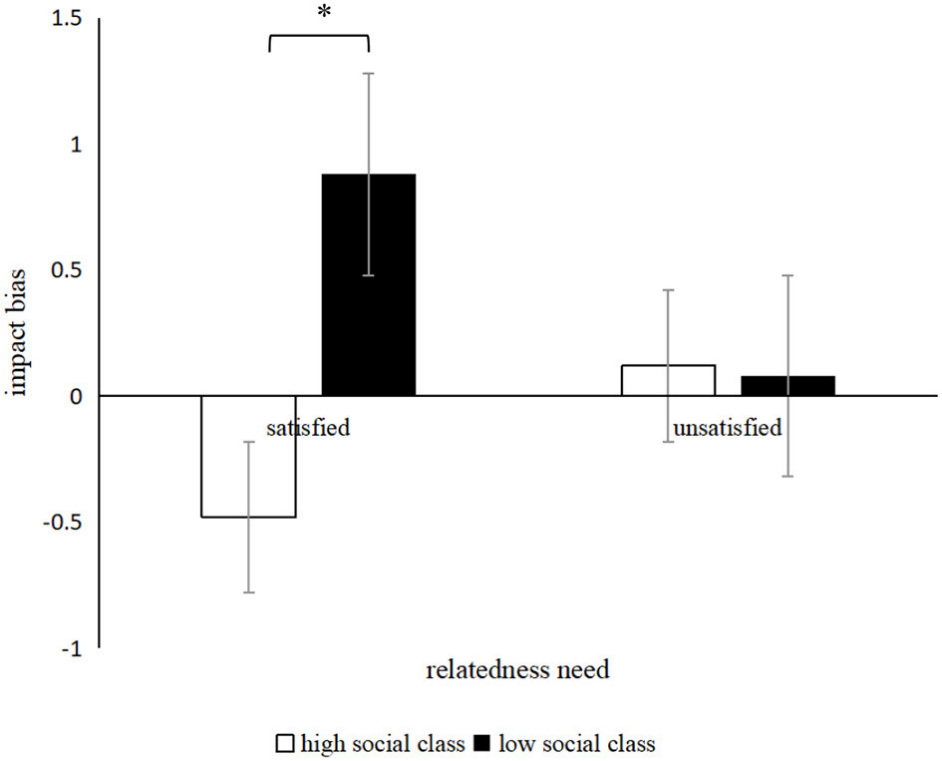
Interactive effect of relatedness need satisfaction and subjective SES on impact bias. ^*^p < 0.05.

According to Gilbert et al. (1998), the difference in affective forecasting minus affective experience was taken as the index of impact bias. As for impact bias, the results of the 2 (subjective SES: high SES vs. low SES) × 2 (relatedness need satisfaction: yes vs. no) ANOVA revealed that the main effect of the subjective SES on impact bias was significant, *F* (1, 119) = 29.76, *p* < 0.001, and η^2^ = 0.20. The impact bias of the lower SES (*M*_lowSES_ = 0.48, *SD* = 0.86) was significantly higher than those of the high SES (*M*_highSES_ = −0.18, *SD* = 0.63). Meanwhile, the main effect of relatedness need was not significant, *F* (1, 119) = 0.67, *p* = 0.42, and η^2^ = 0.01. The interaction of the subjective SES and relatedness need on impact was significant, *F* (1, 119) = 32.81, *p* < 0.001, and η^2^ = 0.22. A simple effect analysis showed that, when the relatedness need was satisfied, the low-SES individual had a greater impact bias than the high-SES individual, *M*_lowSES_ = 0.88, *SD* = 1.02, *M*_highSES_ = −0.48, *SD* = 0.65, *t* (58) = −6.18, 95% CI [−1.81, −0.92], *p* = 0.01 and *d* = −1.62. When the relatedness need was not satisfied, no significant difference was observed between the high-SES individual and the low-SES individuals; *M*_highSES_ = 0.12, *SD* = 0.45, *M*_lowSES_ = 0.08, *SD* = 0.35, *t* (58) = 0.32, 95% CI [−0.17, 0.24], *p* = 0.75 and *d* = 0.08. The interaction is shown in Figure 4.

### 5.3. Discussion

Results of study 3 showed that when relatedness need was satisfied, the impact bias of low-SES individuals was higher than that of high-SES individuals. In contrast, when relatedness need was not satisfied, no significant difference was observed between high-SES and low-SES participants, thus supporting our hypothesis. Given that low-SES individuals attached more importance to relatedness need satisfaction than high-SES individuals, low-SES individuals would have more positive expectations for the experience (i.e., playing the board game) when their relatedness need was satisfied, thus overestimating their own positive emotions more.

## 6. General discussion

### 6.1. Subjective SES and basic psychological needs

The pilot study found that high-SES individuals attached more importance to the need for competence and autonomy than low-SES individuals. Meanwhile, individuals in the low SES attached more importance to relatedness need than those in the high SES. The results suggested that the need strength of basic psychological needs for high-SES individuals and low-SES individuals is different. Ryan and Deci (2000) found that individual differences in the need strength of the three basic psychological needs exist, although self-determination theory suggests that the three basic psychological needs are equivalent with regard to their importance for psychosocial functioning. The current research suggested that SES would be one kind of important individual difference that influences basic psychological need strength. Previous studies showed that individuals of high SES have a strong sense of control over their own lives (Markus and Kitayama, 1991; Lachman and Weaver, 1998; Johnson and Krueger, 2006), while low-SES individuals rely on good interpersonal relationships to obtain abundant social resources (Piff et al., 2010). These differences may lead to the difference in need strength of basic psychological needs.

### 6.2. The effect of basic psychological needs on affective forecasting

The current research found that the main effect of BPNS on affective forecasting was not significant, which was not consistent with our hypothesis. According to the self-determination theory, satisfying basic psychological needs can promote intrinsic motivation (Deci and Ryan, 2000). Compelling evidence shows that greater satisfaction with the needs for competence, autonomy, and relatedness is directly associated with intrinsic motivation and positive emotions in different contexts (e.g., Tang et al., 2020; Stanley et al., 2021). Previous studies demonstrated that impact bias has motivated underpinnings (Morewedge and Buechel, 2013; Pauketat et al., 2016; Geng and Jiang, 2017; Geng et al., 2018). In other words, the intensity of motivation to achieve the expected outcome would increase impact bias in affective forecasting of the expected outcome. However, the current research did not find the main effect of BPNS on impact bias. We thought one possible reason was that the effect of BPNS on affective forecasting was different for high-SES individuals and low-SES individuals, which was confirmed in the moderating analysis of the subjective SES.

### 6.3. The moderating role of subjective SES between BPNS and affective forecasting

The present research demonstrated the moderating effect of undergraduates' subjective SES in the effect of basic psychological needs satisfaction on the impact bias in affective forecasting. When autonomy need or competence need was satisfied, the high-SES individuals overestimated the pleasantness of the product which could satisfy their autonomy need and competence need more than low-SES individuals. In contrast, when relatedness need was satisfied, the low-SES individuals overestimated the pleasantness of the product which could satisfy their relatedness need more than high-SES individuals.

We believe that these findings provide important new insights for affective forecasting research. Based on the basis of the moderating effect of SES in the effect of basic psychological needs satisfaction on the impact bias in affective forecasting, we proposed a *SES—BPNS fit model* of affective forecasting, i.e., when people's preferred basic psychological needs satisfaction fit with their SES, the affective forecasting would be stronger than unfit. To test the *SES—BPNS fit model*, in study 1, we coded the high SES in autonomy need satisfaction condition and low SES in autonomy need non-satisfaction condition as the *fit* condition, and coded the high SES in autonomy need non-satisfaction condition and low SES in autonomy need satisfaction condition as the *non-fit* condition. In study 2, we coded the high SES in competence need satisfaction condition and low SES in competence need non-satisfaction condition as the *fit* condition, and coded the high SES in competence need non-satisfaction condition and low SES in competence need satisfaction condition as the *non-fit* condition. In study 3, we coded the low SES in relatedness need satisfaction condition and high SES in relatedness need non-satisfaction condition as the *fit* condition, and coded the low SES in relatedness need non-satisfaction condition and high SES in relatedness need satisfaction condition as the *non-fit* condition. The independent *t*-test showed that in study 1, *M*_fit_ = 0.70, *SD*_fit_ = 1.12, *M*_nonfit_ = 0.31, *SD*_nonfit_ = 0.96, *t* (118) = 2.06, *p* = 0.04, *d* = 0.38; in study 2, *M*_fit_ =1.76, *SD*_fit_ = 1.83, *M*_nonfit_ = 0.95, *SD*_nonfit_ = 1.76, *t* (118) = 2.48, *p* = 0.01, *d* = 0.46; in study 3, *M*_fit_ =0.50, *SD*_fit_ = 0.87, *M*_nonfit_ = −0.20, *SD*_nonfit_ = 0.59, *t* (118) = 5.14, *p* = 0.01, *d* = 1.01, suggesting the impact bias in fit condition was significantly greater than that in non-fit condition, which was consistent with the *SES—BPNS fit model*.

Even though researchers have investigated numerous individual differences in affective forecasting (Nielsen et al., 2008; Tomlinson et al., 2010; Hoerger et al., 2012), few have shed light on individuals' SES. We think one possible reason for the moderating effect of SES is that when autonomy need or competence need was satisfied, high-SES individuals may have stronger motivation to experience the product which might satisfy their autonomy or competence need and expect higher pleasantness for products, leading to a bigger impact bias. In contrast, when relatedness need was satisfied, low-SES individuals may have stronger motivation to experience the product which might satisfy their relatedness need and increase positive affective expectations for products, leading to bigger impact bias.

The current research also has important implications in practice. It found that high-SES undergraduates overestimated their pleasantness more when autonomy need and competence need were satisfied, while low-SES undergraduates overestimated their pleasantness more when relatedness was satisfied. From the standpoint of marketers, for high-SES consumers, marketers should satisfy their competence need and autonomy need first, and then they would like to buy the products based on their predicted pleasantness, while for low-SES consumers, marketers should satisfy their relatedness need first and then they would like to buy the products based on their predicted pleasantness. However, standing in consumers' shoes, this result suggested high-SES undergraduates not overestimating their pleasantness on those products relevant to autonomy need and competence need satisfaction, and low-SES undergraduates not overestimating their pleasantness on those products relevant to relatedness need satisfaction. However, our study has some limitations. In the present study, we did not examine the interaction effect of the three basic psychological needs on impact bias. Future research can test the interaction effect of different basic psychological needs on affective forecasting, such as high competence need satisfaction with low autonomy need satisfaction or high competence need satisfaction with low relatedness need satisfaction. In addition, future studies are needed to investigate the need strength of high-SES and low-SES individuals directly, such as by the need strength scale. Third, based on the present results, we inferred that motivation may mediate the effect of basic psychological needs satisfaction on affective forecasts; however, we did not measure the motivation directly. Future studies are needed to investigate the mediating role of motivation between BPNS and affective forecasting.

## 7. Conclusion

Overall, the findings of the present research found the moderating role of SES in the effect of basic psychological need satisfaction on affective forecasting. Specifically, for high-SES individuals, the satisfaction of autonomy need/competence need increased the impact bias in affective forecasting on products that are related to autonomy need/competence need more than that for low-SES individuals. Meanwhile, for individuals in the low SES, the satisfaction of relatedness increased the impact bias in affective forecasting on products that are related to relatedness need than high-SES individuals.

## Data availability statement

The original contributions presented in the study are included in the article/supplementary material, further inquiries can be directed to the corresponding author.

## Ethics statement

The studies involving human participants were reviewed and approved by the Academic Ethics Committee of Jing Hengyi School of Education, Hangzhou Normal University. The patients/participants provided their written informed consent to participate in this study.

## Author contributions

FZ, LF, and XG designed the work. LF, MS, and FZ conducted the studies. XJ, LF, and YZ draft the work. XG revised the manuscript. All authors contributed to the article and approved the submitted version.
